# Effect of acute kidney injury on weaning from mechanical ventilation in chronic obstructive pulmonary diseases patients with respiratory failure

**DOI:** 10.1186/s43168-020-00013-2

**Published:** 2020-07-21

**Authors:** Ahmed Gouda El-Gazzar, Mahmoud Mohamad El-Salahy, Tarek Samy Essway, Samar Nasef Mohammed, Marwa Elsayed Elnaggar

**Affiliations:** Faculty of Medicine, Benha University, Benha city, Qalubia Province 13511 Egypt

**Keywords:** Acute kidney injury (AKI), Respiratory failure (RF), Chronic obstructive pulmonary diseases (COPD)

## Abstract

**Background:**

Acute kidney injury (AKI) worsens the outcome in mechanically ventilated patients. The occurrence of AKI may have an effect on weaning from mechanical ventilation (MV). The purpose of this work is to study the effect of AKI on weaning from MV for chronic obstructive pulmonary disease (COPD) patients with respiratory failure (RF). The study included 100 mechanically ventilated COPD patients with RF. Patients were classified into group 1, mechanically ventilated COPD patients with type 2 RF who developed AKI, and group 2, mechanically ventilated COPD patients with type 2 RF, who did not develop AKI.

**Results:**

The age of the studied group ranged between 46 and 80 years, and serum creatinine on admission was within normal. There is a significant difference in a serum creatinine level after 2 days and after 1 week of MV in an intensive care unit (ICU) among AKI cases. There is a significant difference in weaning success between AKI and non-AKI patients with much higher weaning success in non-AKI group. Also, there is a significant reduction in glomerular filtration rate (GFR) in AKI patients after 48 h of admission.

**Conclusions:**

AKI is frequent in COPD patients within 48 h of ICU admission and MV increases the risk of AKI in COPD patients.

## Background

Acute kidney injury (AKI) is the abrupt loss of kidney function, resulting in the retention of urea and other nitrogenous waste products and dysregulation of extracellular volume and electrolytes. It is most easily detected by measurement of the serum creatinine, which is used to estimate the glomerular filtration rate (GFR). AKI was defined as at least one measurement of serum creatinine of > 1.5 mg/dL during the ICU stay [1].

The causes of AKI in the ICU are frequently “multi-factorial,” and in most cases, AKI develops from a combination of hypovolemia, sepsis, nephrotoxins, and hemodynamic disturbances [2].

AKI can increase pulmonary vascular permeability and downregulate ion channels critical for fluid absorption in the lungs, leading to pulmonary inflammation, hemorrhage, septal edema, and apoptosis. The occurrence of lung injury can also affect the kidneys. Mechanical ventilation (MV) causes hemodynamic abnormalities, which can, in turn, affect renal perfusion by reducing cardiac output and stimulating hormonal and sympathetic pathways [3].

Besides that, the presence of AKI in critically ill patients like those with respiratory failure frequently contributes to depression of mental status and accumulation of several drug metabolites, which can further compromise the level of consciousness [4].

## Aim of the work

The aim of this work was to study the effect of acute kidney injury on weaning from mechanical ventilation for COPD patients with respiratory failure including successfulness, duration of weaning, and the length of patients stay in the respiratory care unit.

## Methods

The prospective study included 100 mechanically ventilated COPD patients with respiratory failure admitted at the respiratory intensive care unit of Benha University Hospitals during the period from September 2016 to May 2018.

Acute exacerbation of COPD with respiratory failure presented with one of the following:

- Respiratory acidosis (PaCO_2_ ≥ 45 mmHg and arterial pH ≤ 7.35)
- Severe dyspnea with respiratory muscle fatigue
- Persistent hypoxemia with oxygen supplementation

These patients failed to respond to NIV (non-invasive ventilation), presented with respiratory or cardiac arrest, have decreased conscious level or hemodynamically unstable and indicated for invasive mechanical ventilation [5].

Patients were classified into 2 groups:

- Group 1: mechanically ventilated COPD patients (diagnosed according to GOLD 2017) with type 2 RF who developed acute kidney injury (AKI).
- Group 2: mechanically ventilated COPD patients with type 2 RF but not developed AKI.

AKI was defined according to KDIGO as any of the following [6]:

- Increase in serum creatinine by 0.3 mg/dL or more within 48 h
- Increase in serum creatinine to 1.5 times baseline or more within the last 7 days

Inclusion criteria:

- COPD patients with type 2 respiratory failure plus AKI after 48 h of starting mechanical ventilation (MV).
- Invasive MV for > 48 h

Exclusion criteria:

Patients with (chronic kidney injury, respiratory failure due to causes other than COPD and who receiving drugs altering renal function)

All patients subjected to the following:

1. Full history taking and clinical examination
2. Plain chest X-ray
3. Routine laboratory investigations (CBC, liver, and kidney function tests)
4. Arterial blood gases (ABG) using Sensa Core’s ST-200 CC Blood Gas Analyzer (Sensa Core Medical Instrumentation Pvt. Ltd. Plot No. 3, EPIP Zone, Pashamylaram, Sangareddy District, Telangana, 502307 Hyderabad, India.)
5. Assessment for AKI
  A. Serum (creatinine, Na, and K)
  B. Estimation of GFR: through the following equations:
    **CKD-EPI** (chronic kidney disease epidemiology collaboration); GFR = 141 × min (S_cr_/κ, 1) ^α^ × max (S_cr_/κ, 1)^-1.209^ × 0.993^age^ × 1.018 [if female] × 1.159 [if black] [7].
      **NB**: where S_cr_ is serum creatinine in mg/dL, *κ* is 0.7 for females and 0.9 for males, *α* is − 0.329 for females and − 0.411 for males, min indicates the minimum of S_cr_/κ or 1, and max indicates the maximum of S_cr_/*κ* or 1.
    **MDRD** (the modification of diet in renal disease): GFR (mL/min/1.73 m^2^) = 175 × (Scr)-1.154 × (age) − 0.203 × (0.742 if female) × (1.212 if black) [8].
6. APACHE II score calculation [9]: The point score is calculated (Table 1) from a patient’s age and 12 routine physiological measurements measured during the first 24 h after admission including [A-aDO_2_ or PaO_2_ (depending on FiO_2_), temperature (rectal), mean arterial pressure, pH arterial, heart rate, respiratory rate, sodium (serum), potassium (serum), creatinine, hematocrit, white blood cell count, Glasgow coma scale].

Ventilator setting for both groups [10]:

- Starting with assist control mode ventilation with a volume target
- Fraction of inspired oxygen (FiO_2_) was set to target SpO_2_ of 88–92%
- Tidal volume (VT) 6–8 ml/kg, respiratory rate (RR) 12–14/min, inspiratory: expiratory (I: E) ratio = 1:3 or more, flow rate 80–100 L/min, peak inspiratory pressure (PIP) of < 40–45 cm H_2_O and Pplat < 30 cm H_2_O was acceptable
- PEEP setting and inspiratory pressure support started at 5 cm H_2_O and 10 cm H_2_O, respectively.

## Ethics approval and consent

The Research Ethics Committee at the Faculty of Medicine, Benha University, has approved the study, and all patients provided written informed consent before participation

## Statistical analysis

All data were collected, tabulated, and statistically analyzed using STATA/SE version 11.2 for Windows (STATA Corporation, College Station, Texas). Continuous data were expressed as the mean ± SD and range, and categorical data were expressed as a number and percentage. Student *t* test (*t*) and Mann-Whitney test (*z*) were used to compare two groups of normally and non-normally distributed data, respectively. One-way analysis of variance (ANOVA; *F*) and Kruskal-Wallis test (*x*^2^) were used to compare more than two groups. Percent of categorical variables was compared using the chi-square (χ^2^) test and Fisher’s exact test as appropriate. Statistical significance was accepted at *P* value < 0.05 (S). A *P* value < 0.001 was considered highly significant (HS) while a *P* value > 0.05 was considered non-significant [11].

## Results

**Fig. 1:**
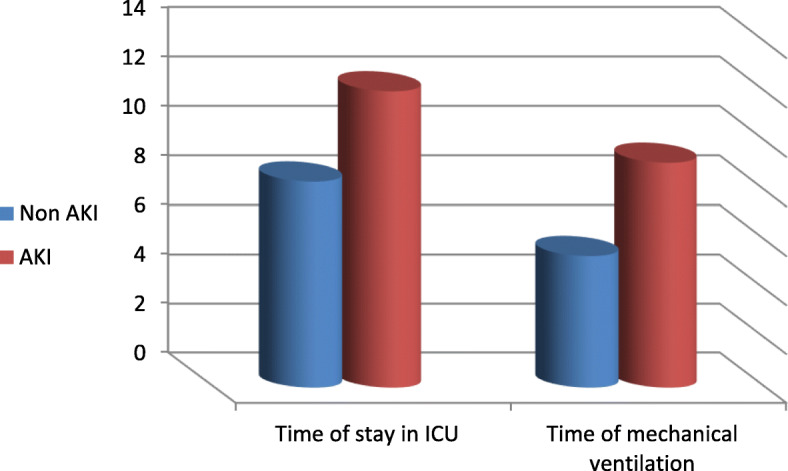
Time of stay in ICU and on mechanical ventilation (in days) between patients with COPD with and without AKI

The age of the studied group is between 46 and 80 years with males (86%) are more than females (14%), and serum creatinine on admission was within normal (0.9 ± 0.2). Serum creatinine was significantly higher in COPD patients with AKI 48 h and 1 week following mechanical ventilation (Table 2). Weaning success in COPD patients was related to lower APACHE II score, low PEEP used during mechanical ventilation, less days in ICU, and less days on mechanical ventilation (Table 3). Also, the presence of AKI increased the chance of weaning difficulty and death (Table 4). APACHE II score was found to be significantly higher in COPD patients with AKI compared with patients without AKI. Also, GFR decreased 48 h following ICU admission in COPD patients with AKI. It was found also that, COPD with AKI required higher PEEP, more days on mechanical ventilation and more ICU stay while other ventilator parameters did not show a significant difference between COPD patients with or without AKI (Table 5, Fig. 1). About 27.59% of COPD patients with AKI showed increased echogenicity of the kidney while 6 patients developed bilateral grade I nephropathy (Table 6).

**Table 1:** Interpretation of score

Score	0–4	5–9	10–14	15–19	20–24	25–29	30–34	> 34
Death rate (%)	4	8	15	25	40	55	75	85

## Discussion

Many COPD patients have latent kidney injury (KI) that manifested on MV. According to Cerda et al., the prevalence of AKI within the overall COPD cohort was 128/100,000 person-years, the prevalence of concomitant AKI at exacerbation changed into 1.9%, and the mortality rate in patients with AKI at exacerbation was 521/1000 person-years [12].

Regarding serum creatinine level in the studied groups, this study showed that there was a significant difference in serum creatinine levels after 2 days and after 1 week of MV in ICU among the 1st group than non-AKI group (Table 2). Vieira et al. studied 140 patients in the intensive care unit (ICU), 93 with AKI and 47 controls and demonstrated that ≥ 85% increase in baseline serum creatinine level in AKI cases [13].

**Table 2:** Comparison of serum creatinine level in the studied groups on admission, after 2 days and after 1 week of starting mechanical ventilation

Serum creatinine (mg/dl)	Non AKI (no. = 42) mean ± SD	AKI (no. = 58) mean ± SD	Test	*P* value
**On admission**	0.84 ± 0.18	0.94 ± 0.21	*t* = 1.79	0.08
**After 48 h of MV**	0.82 ± 0.15	2.0 ± 0.27	*t* = 17.80	< 0.001(HS)
**After 1 week of MV**	0.89 ± 0.17	2.05 ± 0.47	*t* = 10.78	< 0.001 (HS)

*AKI* acute kidney injury, *MV* mechanical ventilation

This indicates that there is a rapid decline in renal function from baseline occurring over several hours resulting in the accumulation of nitrogenous wastes such as urea and creatinine [14].

In meta-analysis review by Van den Akker et al., they found that the pooled odds ratio for the overall effect of MV on AKI was 3.16 to 4.18 and that all subgroups showed that MV increases the risk of AKI. They concluded that invasive MV is associated with a threefold increase in the odds of developing AKI and various Vt or PEEP settings do not modify the risk [15].

In this study, there was a significant relation between weaning success and the APACHE II score as well as the time of stay in ICU. Patients with lower APACHE II score get better weaning results, less time of MV, less need to increase PEEP, and less time of stay in ICU. Difficult weaning and dead patients showed significant higher levels of PEEP needed, longer duration of ICU stay, and longer duration of MV in days (Table 3).

**Table 3:** Relation between weaning success and parameters of admission in ICU including APACHE II score

Variable	Weaning success							
	Weaned and discharged (no. = 72)		Died (mostly hypoxic arrest) (no. = 22)		Difficult weaning (no. = 6)		Test	*P*
	Mean ± SD	Range	Mean ± SD	Range	Mean ± SD	Range		
APACHE II score	17.36 ± 4.73	11–28	25.36 ± 6.44	15–34	28.33 ± 2.89	25–30	*F* = 14.97	< 0.001 (HS)
Mechanical ventilation parameter (PEEP)	5 ± 0	5	6.36 ± 2.01	5–10	9.33 ± 2.08	7–11	*X*^2^ = 10.53	0.005 (S)
Time of stay in ICU in days	8.97 ± 1.98	7–14	10.73 ± 2.53	7–15	28 ± 2	26–30	*X*^2^ = 13.07	0.001 (S)
Time of mechanical ventilation in days	6.03 ± 2.05	4–15	8.73 ± 2.65	6–13	21.67 ± 3.78	19–26	*X*^2^ = 18.78	< 0.001 (HS)

*ICU* intensive care unit, *APACHE II* acute physiology and chronic health evaluation II, *PEEP* positive end-expiratory pressure

Vieira et al. showed that the length of ICU stays and ICU mortality rate were significantly greater in the AKI patients. After adjusting for the APACHE II score, it was higher among AKI patients (24 ± 8.1 versus 20 ± 10; *p* = 0.04) [13].

In another study done by Clermont et al., it was not clear whether the outcome is related to respiratory complications or duration of MV and whether even a mild AKI would be still associated with a poor outcome. They were able to demonstrate a relationship between the development of AKI in the ICU and the increase in mortality rate [16].

These results assume that reversible factors have to be optimized (e.g., renal, metabolic) that the eventual clinical outcome of patients with prolonged weaning failure will depend on the correction of the underlying disease [17].

In the current study, there was a higher percentage of weaning success in non-AKI than AKI patients. Also, difficult weaning and dead patients were significantly higher in AKI patients, meaning that the development of AKI inversely affects weaning from MV (Table 4).

**Table 4:** Comparison of weaning success between AKI and non-AKI cases

Weaning success	Non-AKI (no. = 42)		AKI (no. = 58)		*P*
	No.	%	No.	%	
Weaned and discharged	38	90.48	34	58.62	0.04 (S)
Died	4	9.52	18	31.03	
Difficult weaning	0	0.0	6	10.34	

*AKI* acute kidney injury

Rothaar et al. showed that the duration of MV and the time spent in weaning was significantly longer in patients with AKI. The rate of weaning failure in their study is similar to known literature, around (20%), although the weaning failure in the AKI patients was not significantly different. The exact role of AKI in lengthening the duration of weaning is not clear, but they suggested that the findings can be partially explained by the interactions of kidney injury and respiratory function [18].

The present study showed that APACHE II score was significantly higher in AKI cases than non-AKI cases reflecting the impact of AKI on the outcome of ICU admission (Table 5).

**Table 5:** Relation between kidney injury and APACHE II score, MV parameters, duration of ICU stay, and GFR among the studied groups

Variable		Non-AKI (no. = 42) Mean ± SD	AKI (no. = 58) Mean ± SD	Test	*P*
APACHE II score		17.19 ± 4.79	21.65 ± 6.77	*Z* = 2	0.04
Mechanical ventilation parameters	PEEP	5 ± 0	5.96 ± 1.88	*Z* = 2.39	0.02(S)
	Vt ml/kg	7.05 ± 0.74	7.03 ± 0.73	*Z* = 0.05	0.9
	RR	12 ± 0	12.38 ± 0.73	*Z* = 1.4	0.15
	PaO_2_/FiO_2_	236.3 ± 33.73	233.9 ± 23.05	*Z* = 0.69	0.49
	Inspiratory pressure	10.97 ± 1.88	11.05 ± 1.6	*Z* = − 0.59	0.56
Time of stay in ICU in days		8.38 ± 1.36	12.03 ± 6.01	*Z* = 3.04	0.002(S)
Time of mechanical ventilation in days		5.38 ± 1.16	9.14 ± 5.15	*Z* = 3.90	< 0.001(HS)
Time of starting MV in days		1.71 ± 0.56	1.9 ± 0.82	*t* = 0.88	0.38
GFR after 48 h of admission (ml/min/1.73 m^2^)		105.09 ± 11.36	41.69 ± 5.75	*t* = 25.88	< 0.001(HS)

*APACHE II* acute physiology and chronic health evaluation II, *AKI* acute kidney injury, *MV* mechanical ventilation, *GFR* glomerular fiteration rate, *Vt* tidal volume, *RR* respiratory rate, PaO_2_/FiO_2_ arterial oxygen tension/fraction of inspired oxygen, *ICU* intensive care unit, *AKI* acute kidney injury, *PEEP* positive end-expiratory pressure

Esteban et al. studied 23 medical/surgical ICU patients with similar criteria for our patients. APACHE II score did not differ between groups, but patients who developed AKI were more severely ill in the long term. However, after adjusting for severity, they still could identify worsening of renal function, defined by an increase of 85% in SCr, and the occurrence of oliguria, as independent factors associated with prolonged time for weaning. They concluded that in mechanically ventilated older patients, AKI was one of the most important negative predictive factors for ICU outcomes [19].

In this study, it was found that AKI results in a significant increase in required PEEP, ICU stay, and time of MV in days. There is no significant difference between AKI and non-AKI patients as regards time before starting MV, reflecting the effect of AKI on all these parameters (Table 5, Fig. 1).

Amato et al. showed that AKI patients who were weaned from MV indeed used higher PEEPs [20].

Santos and Magro demonstrate the effect PEEP in critically ill patients which can result in altered kidney function in patients in ICU [21]. This is also in agreement with Mehta et al. who explained that a high rate of oliguria and the lower urinary volume in AKI patients probably contributed to positive net fluid balance, confirming recent findings on the effect of fluid balance on weaning [22].

Koyner and Murray showed that there is a bidirectional relationship between the AKI and ALI and shows that injury to one organ may initiate and aggravate an injury to the other. They suggested that this phenomenon might be termed ventilator-induced kidney injury (VIKI) [23].

In this study, there was a significant reduction in GFR in AKI patients after 48 h of admission than in non-AKI patients, denoting that AKI develops early in susceptible patients after ICU admission or MV start (Table 5).

Elmahallawy and Qora reported that the mean estimated GFR in their COPD group was 75.20 ± 35.78 ml/min/1.73 m^2^, while for the control group, it was 92.04 ± 25.54 ml/min/1.73 m^2^ with a highly significant decrease in GFR in COPD group (*p* < 0.01). The lower GFR in COPD group in their study was attributed to old age with more severe airflow limitation, and 68% of them had chronic respiratory failure, which points to the susceptibility of COPD patients to AKI [24].

Our study found a significant difference in sonographic findings between the two groups, with increased echogenicity, bilateral grade 1 nephropathy in AKI patients, than non-AKI patients, denoting organic kidney affection not just with functional impairment (Table 6).

**Table 6:** Relation between sonographic findings and kidney injury among the studied groups

U/S finding after 48 h of admission	Non-AKI (no. = 42)		AKI (no. = 58)		*P*
	No.	%	No.	%	
Normal echogenic kidneys	42	100.0	36	62.07	0.002 (S)
Slight increased echogenicity of the kidneys	0	0.0	16	27.59	
Bilateral grade 1 nephropathy	0	0.0	6	10.34	

*U/S* ultrasonography, *AKI* acute kidney injury

Grade I hyperechogenicity was the most important universal finding in acute kidney injury differentiating it from chronic renal failure in a study by Ozmen et al. to evaluate ultrasound in differentiating acute from chronic kidney disease [25].

## Conclusion

AKI is frequent in COPD patients within 48 h of ICU admission and mechanical ventilation increase the risk of AKI in COPD patients. Also, assessment of GFR in COPD patients is recommended for early detection and monitoring of renal function abnormalities.

## Data Availability

Not applicable.

## Abbreviations

AKI: Acute kidney injury
MV: Mechanical ventilation
COPD: Chronic obstructive pulmonary diseases
RF: Respiratory failure
ICU: Intensive care unit
GFR: Glomerular filtration rate
PaCo_2_: Arterial tension of carbon dioxide
PH: Power of hydrogen
NIV: Non-invasive ventilation
GOLD: Global initiative for chronic obstructive lung disease
KDIGO: Kidney disease improving global outcomes
CBC: Complete blood count
ABG: Arterial blood gas
Na: Sodium
K: Potassium
CKD-EPI: Chronic kidney disease epidemiology collaboration
MDRD: Modification of diet in renal disease
APACHE II: Acute physiology and chronic health evaluation II
PaO_2_: Arterial oxygen tension
FiO_2_: Fraction of inspired oxygen
Vt: Tidal volume
RR: Respiratory rate
I:E: Inspiratory/expiratory
PIP: Peak inspiratory pressure
Pplat: Plateau pressure
PEEP: Positive end-expiratory pressure
Scr: Serum creatinine
ALI: Acute lung injury
VIKI: Ventilator-induced kidney injury
